# Supplementary material for: Complete Chloroplast Genome of Rhipsalis baccifera, the only Cactus with Natural Distribution in the Old World: Genome Rearrangement, Intron Gain and Loss, and Implications for Phylogenetic Studies
Source: Plants (Basel). 2020 Jul 31;9(8):979. doi: 10.3390/plants9080979 (PMC7464518; doi:10.3390/plants9080979)
Supplement: Supplementary file 1 [file plants-09-00979-s001.zip › Figure S2.docx]

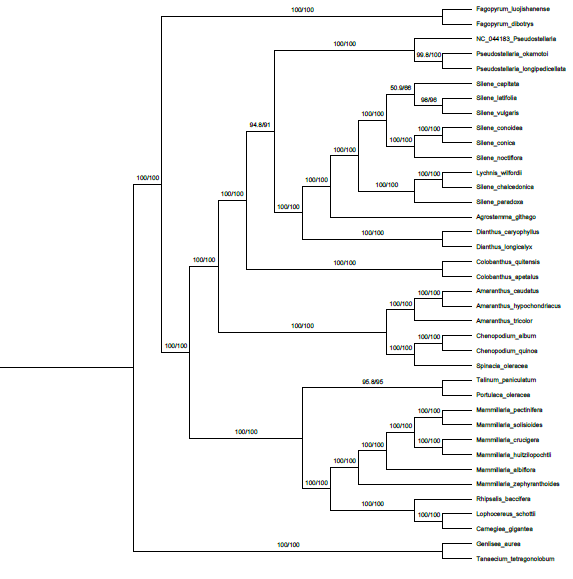


**Figure S2.** Phylogenetic analysis of thirty six species order Caryophyllales using using maximum likelihood (ML) and GTR+F+G4 substitution model based on their complete chloroplast genomes. Two outgroups from order Lamiales were used. The numbers on the tree branches represent bootstrap values.
