# Supplementary material for: Complete Chloroplast Genome of Rhipsalis baccifera, the only Cactus with Natural Distribution in the Old World: Genome Rearrangement, Intron Gain and Loss, and Implications for Phylogenetic Studies
Source: Plants (Basel). 2020 Jul 31;9(8):979. doi: 10.3390/plants9080979 (PMC7464518; doi:10.3390/plants9080979)
Supplement: Supplementary file 1 [file plants-09-00979-s001.zip › Figure S3.docx]

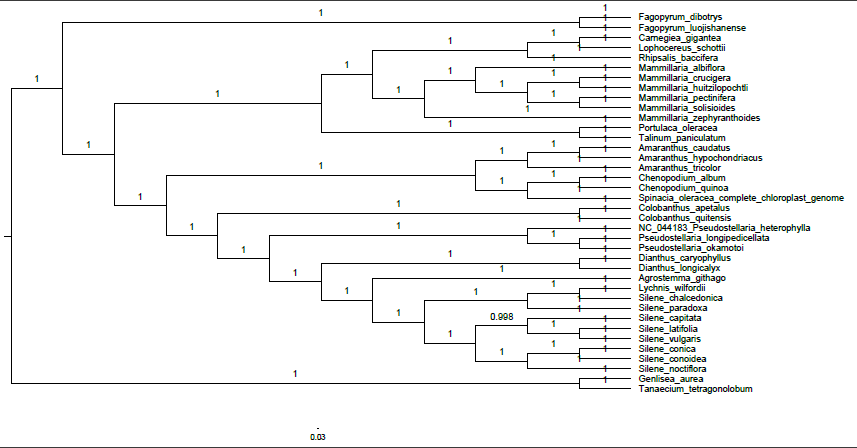


**Figure S3.** Phylogenetic analysis of thirty six species order Caryophyllales using using Bayesian method and GTR+G+F model substitution model based on their complete chloroplast genomes. Two outgroups from order Lamiales were used.
