# Supplementary material for: Complete Chloroplast Genome of Rhipsalis baccifera, the only Cactus with Natural Distribution in the Old World: Genome Rearrangement, Intron Gain and Loss, and Implications for Phylogenetic Studies
Source: Plants (Basel). 2020 Jul 31;9(8):979. doi: 10.3390/plants9080979 (PMC7464518; doi:10.3390/plants9080979)
Supplement: Supplementary file 1 [file plants-09-00979-s001.zip › Table S1.docx]

**Table S1:** Complete chloroplast genomes used in phylogenetic analysis

| Species Name | NCBI Accession Number | Family |
| --- | --- | --- |
| *Agrostemma githago* | NC_023357 | Caryophyllaceae |
| *Lychnis wilfordii* | NC_035225 | Caryophyllaceae |
| *Silene chalcedonica* | NC_023359 | Caryophyllaceae |
| *Silene conica* | NC_016729 | Caryophyllaceae |
| *Silene conoidea* | NC_023358 | Caryophyllaceae |
| *Silene noctiflora* | NC_016728 | Caryophyllaceae |
| *Silene paradoxa* | NC_023360 | Caryophyllaceae |
| *Silene capitata* | NC_035226 | Caryophyllaceae |
| *Silene latifolia* | NC_016730 | Caryophyllaceae |
| *Silene vulgaris* | NC_016727 | Caryophyllaceae |
| *Dianthus caryophyllus* | NC_039650 | Caryophyllaceae |
| *Dianthus longicalyx* | KM668208 | Caryophyllaceae |
| *Pseudostellaria heterophylla* | NC_044183 | Caryophyllaceae |
| *Pseudostellaria longipedicellata* | MH373593 | Caryophyllaceae |
| *Pseudostellaria okamotoi* | MH879018 | Caryophyllaceae |
| *Colobanthus apetalus* | MF687919 | Caryophyllaceae |
| *Colobanthus quitensis* | NC_028080 | Caryophyllaceae |
| *Amaranthus caudatus* | NC_040143 | Amaranthaceae |
| *Amaranthus hypochondriacus* | MG836505 | Amaranthaceae |
| *Amaranthus tricolor* | KX094399 | Amaranthaceae |
| *Chenopodium album* | NC_034950 | Chenopodiaceae |
| *Chenopodium quinoa* | KU255732 | Chenopodiaceae |
| *Spinacia oleracea* | AJ400848 | Chenopodiaceae |
| *Carnegiea gigantea* | NC_027618 | Cactaceae |
| *Lophocereus schottii* | NC_041727 | Cactaceae |
| *Mammillaria albiflora* | MN517610 | Cactaceae |
| *Mammillaria crucigera* | MN517613 | Cactaceae |
| *Mammillaria huitzilopochtli* | MN517612 | Cactaceae |
| *Mammillaria pectinifera* | MN519716 | Cactaceae |
| *Mammillaria solisioides* | MN518341 | Cactaceae |
| *Mammillaria zephyranthoides* | MN517611 | Cactaceae |
| *Rhipsalis baccifera* |  | Cactaceae |
| *Portulaca oleracea* | NC_036236 | Portulacaceae |

| Continued table S1 |  |  |
| --- | --- | --- |
| *Talinum paniculatum* | MG710385 | Talinaceae |
| *Fagopyrum dibotrys* | NC_037705 | Polygonaceae |
| *Fagopyrum luojishanense* | NC_037706 | Polygonaceae |
| *Genlisea aurea* | MF593121 | Lentibulariaceae |
| *Tanaecium tetragonolobum* | KR534325 | Bignoniaceae |
