# Supplementary material for: Complete Chloroplast Genome of Rhipsalis baccifera, the only Cactus with Natural Distribution in the Old World: Genome Rearrangement, Intron Gain and Loss, and Implications for Phylogenetic Studies
Source: Plants (Basel). 2020 Jul 31;9(8):979. doi: 10.3390/plants9080979 (PMC7464518; doi:10.3390/plants9080979)
Supplement: Supplementary file 1 [file plants-09-00979-s001.zip › Table S2.docx]

**Table S2:** Codon usage in the chloroplast genome of *Rhipsalis baccifera*

| Amino acid | Codon | Count | RSCU | *tRNA* | Amino acid | Codon | Count | RSCU | *tRNA* |
| --- | --- | --- | --- | --- | --- | --- | --- | --- | --- |
| Phe(f) | UUU(F) | 813 | 1.37 |  | Tyr | UAU(Y) | 566 | 1.58 |  |
| Phe | UUC(F) | 372 | 0.63 | *trnF-GAA* | Tyr | UAC(Y) | 149 | 0.42 | *trnY-GUA* |
| Leu | UUA(L) | 653 | 1.88 | *trnL-UAA* | Stop | UAA(*) | 38 | 1.58 |  |
| Leu | UUG(L) | 429 | 1.24 | *trnL-CAA* | Stop | UAG(*) | 18 | 0.75 |  |
| Leu | CUU(L) | 458 | 1.32 |  | His | CAU(H) | 361 | 1.42 |  |
| Leu | CUC(L) | 120 | 0.35 |  | His | CAC(H) | 148 | 0.58 | *trnH-GUG* |
| Leu | CUA(L) | 293 | 0.84 | *trnL-UAG* | Gln | CAA(Q) | 611 | 1.57 | *trnQ-UUG* |
| Leu | CUG(L) | 131 | 0.38 |  | Gln | CAG(Q) | 166 | 0.43 |  |
| Ile | AUU(I) | 831 | 1.54 |  | Asn | AAU(N) | 722 | 1.5 |  |
| Ile | AUC(I) | 288 | 0.53 | *trnI-GAU* | Asn | AAC(N) | 241 | 0.5 | *trnN-GUU* |
| Ile | AUA(I) | 500 | 0.93 | *trnI-UAU* | Lys | AAA(K) | 941 | 1.47 | *trnK-UUU* |
| Met | AUG(M) | 449 | 1 | *trn(f)M-CAU* | Lys | AAG(K) | 337 | 0.53 |  |
| Val | GUU(V) | 412 | 1.47 |  | Asp | GAU(D) | 688 | 1.55 |  |
| Val | GUC(V) | 133 | 0.47 | *trnV-GAC* | Asp | GAC(D) | 198 | 0.45 | *trnD-GUC* |
| Val | GUA(V) | 409 | 1.46 | *trnV-UAC* | Glu | GAA(E) | 863 | 1.51 | *trnE-UUC* |
| Val | GUG(V) | 170 | 0.6 |  | Glu | GAG(E) | 277 | 0.49 |  |
| Ser | UCU(S) | 399 | 1.63 |  | Cys | UGU(C) | 166 | 1.49 |  |
| Ser | UCC(S) | 244 | 1 | *trnS-GGA* | Cys | UGC(C) | 57 | 0.51 | *trnC-GCA* |
| Ser | UCA(S) | 280 | 1.15 | *trnS-UGA* | Stop | UGA(*) | 16 | 0.67 |  |
| Ser | UCG(S) | 144 | 0.59 |  | Trp | UGG(W) | 353 | 1 | *trnW-CCA* |
| Pro | CCU(P) | 323 | 1.45 |  | Arg | CGU(R) | 301 | 1.39 | *trnR-ACG* |
| Pro | CCC(P) | 221 | 0.99 | *trnP-GGG* | Arg | CGC(R) | 82 | 0.38 |  |
| Pro | CCA(P) | 238 | 1.06 | *trnP-UGG* | Arg | CGA(R) | 287 | 1.32 |  |
| Pro | CCG(P) | 112 | 0.5 |  | Arg | CGG(R) | 86 | 0.4 |  |
| Thr | ACU(T) | 412 | 1.64 |  | Ser | AGU(S) | 301 | 1.23 |  |
| Thr | ACC(T) | 184 | 0.73 | *trnT-GGU* | Ser | AGC(S) | 98 | 0.4 | *trnS-GCU* |
| Thr | ACA(T) | 309 | 1.23 | *trnT-UGU* | Arg | AGA(R) | 386 | 1.78 | *trnR-UCU* |
| Thr | ACG(T) | 101 | 0.4 |  | Arg | AGG(R) | 160 | 0.74 |  |
| Ala | GCU(A) | 496 | 1.75 |  | Gly | GGU(G) | 458 | 1.33 |  |
| Ala | GCC(A) | 178 | 0.63 |  | Gly | GGC(G) | 148 | 0.43 | *trnG-GCC* |
| Ala | GCA(A) | 341 | 1.21 |  | Gly | GGA(G) | 545 | 1.58 |  |
| Ala | GCG(A) | 116 | 0.41 |  | Gly | GGG(G) | 228 | 0.66 |  |
