# Supplementary material for: Complete Chloroplast Genome of Rhipsalis baccifera, the only Cactus with Natural Distribution in the Old World: Genome Rearrangement, Intron Gain and Loss, and Implications for Phylogenetic Studies
Source: Plants (Basel). 2020 Jul 31;9(8):979. doi: 10.3390/plants9080979 (PMC7464518; doi:10.3390/plants9080979)
Supplement: Supplementary file 1 [file plants-09-00979-s001.zip › Table S3.docx]

Table S3: Table showing the frequency of repeats and their location in chloroplast genome of *Rhipsalis baccifera*

| No. | Size (bp) | Type | Repeat 1 Start | Repeat 1 Location | Repeat 2 Location | Repeat 2 Start | | Location | |
| --- | --- | --- | --- | --- | --- | --- | --- | --- | --- |
| 1 | 122 | F | 46895 | *IGS (trnT-GGU, trnE-UUC)* | *IGS (trnH-GUG, psbA)* | 122211 | | LSC | |
| 2 | 119 | F | 88584 | *IGS (trnL-CAA, ycf 1)* | *IGS (trnL-CAA, ycf 1)* | 88627 | | IRb | |
| 3 | 119 | P | 88586 | *IGS (trnL-CAA, ycf 1)* | *IGS (rps7, trnL-CAA)* | 114763 | | IRb, IRa | |
| 4 | 119 | P | 88629 | *IGS (trnL-CAA, ycf 1)* | *IGS (rps7, trnL-CAA)* | 114806 | | IRb, IRa | |
| 5 | 119 | F | 114764 | *IGS (rps7, trnL-CAA)* | *IGS (rps7, trnL-CAA)* | 114807 | | IRa | |
| 6 | 105 | F | 79912 | *rps19* | *rps19* | 79933 | | LSC | |
| 7 | 89 | F | 88491 | *IGS (trnL-CAA, ycf 1)* | *IGS (trnL-CAA, ycf 1)* | 88620 | | IRb | |
| 8 | 89 | P | 88492 | *IGS (trnL-CAA, ycf 1)* | *IGS (rps7, trnL-CAA)* | 114,801 | | IRb, IRa | |
| 9 | 89 | P | 88621 | *IGS (trnL-CAA, ycf 1)* | *IGS (rps7, trnL-CAA)* | 114930 | | IRb, IRa | |
| 10 | 89 | F | 114802 | *IGS (rps7, trnL-CAA)* | *IGS (rps7, trnL-CAA)* | 114931 | | IRa | |
| 11 | 84 | F | 88,490 | *IGS (trnL-CAA, ycf 1)* | *IGS (trnL-CAA, ycf 1)* | 88,662 | | IRb | |
| 12 | 84 | P | 88,490 | *IGS (trnL-CAA, ycf 1)* | *IGS (rps7, trnL-CAA)* | 114763 | | IRb, IRa | |
| 13 | 84 | P | 88662 | *IGS (trnL-CAA, ycf 1)* | *IGS (rps7, trnL-CAA)* | 114935 | | IRb, IRa | |
| 14 | 84 | F | 114763 | *IGS (rps7, trnL-CAA)* | *IGS (rps7, trnL-CAA)* | 114935 | | IRa | |
| 15 | 81 | F | 88498 | *IGS (trnL-CAA, ycf 1)* | *IGS (trnL-CAA, ycf 1)* | 88584 | IRb | |  |
| 16 | 81 | P | 88498 | *IGS (trnL-CAA, ycf 1)* | *IGS (rps7, trnL-CAA)* | 114844 | | IRb, IRa | |
| 17 | 81 | P | 88584 | *IGS (trnL-CAA, ycf 1)* | *IGS (rps7, trnL-CAA)* | 114930 | IRb, IRa | |  |
| 18 | 81 | F | 114844 | *IGS (rps7, trnL-CAA)* | *IGS (rps7, trnL-CAA)* | 114930 | IRa | |  |
| 19 | 84 | F | 79911 | *rps19* | *rps19* | 79953 | LSC | |  |
| 20 | 76 | F | 88584 | *IGS (trnL-CAA, ycf 1)* | *IGS (trnL-CAA, ycf 1)* | 88670 | IRb | |  |
| 21 | 76 | P | 88584 | *IGS (trnL-CAA, ycf 1)* | *IGS (rps7, trnL-CAA)* | 114763 | IRa, IRb | |  |
| 22 | 76 | P | 88670 | *IGS (trnL-CAA, ycf 1)* | *IGS (rps7, trnL-CAA)* | 114849 | IRa, IRb | |  |
| 23 | 76 | F | 114763 | *IGS (rps7, trnL-CAA)* | *IGS (rps7, trnL-CAA)* | 114849 | IRa | |  |
| 24 | 83 | P | 89978 | *ycf 1* | *IGS (rps7, trnL-CAA)* | 113514 | IRb, IRa | |  |
| 25 | 82 | F | 65576 | *rps18* | *rps18* | 65600 | LSC | |  |
| 26 | 80 | F | 65568 | *rps18* | *rps18* | 65592 | LSC | |  |
| 27 | 79 | F | 54207 | *IGS (trnM-CAU, accD)* | *IGS (trnM-CAU, accD)* | 54262 | LSC | |  |
| 28 | 78 | F | 65345 | *rps18* | *rps18* | 65600 | LSC | |  |
| 29 | 70 | F | 89907 | *ycf1* | *ycf1* | 89973 | IRb | |  |
| 30 | 76 | F | 29745 | *IGS (trnD-GUC, trnF-GAA)* | *IGS (rrn16, trnV-GAC)* | 109806 | LSC, SSC | |  |
| 31 | 75 | F | 29754 | *IGS (trnD-GUC, trnF-GAA)* | *IGS (rrn16, trnV-GAC)* | 109815 | LSC, SSC | |  |
| 32 | 68 | F | 55463 | *accD* | *accD* | 55523 | LSC | |  |
| 33 | 64 | F | 65489 | *rps18* | *rps18* | 65510 | LSC | |  |
| 34 | 71 | F | 65611 | *rps18* | *rps18* | 65635 | LSC | |  |
| 35 | 63 | F | 79911 | *rps19* | *rps19* | 79974 | LSC | |  |
| 36 | 66 | F | 65361 | *rps18* | *rps18* | 65616 | LSC | |  |
| 37 | 58 | F | 54224 | *IGS (trnM-CAU, accD)* | *IGS (trnM-CAU, accD)* | 54279 | LSC | |  |
| 38 | 64 | F | 65430 | *rps18* | *rps18* | 65565 | LSC | |  |
| 39 | 54 | P | 8268 | *IGS (clpP, trnG-UCC)* | *IGS (rps12,psbB)* | 67443 | LSC | |  |
| 40 | 48 | F | 55483 | *accD* | *accD* | 55543 | LSC | |  |
| 41 | 48 | F | 55600 | *accD* | *accD* | 55624 | LSC | |  |
| 42 | 57 | F | 89701 | *ycf1* | *ycf1* | 89719 | IRb | |  |
| 43 | 57 | P | 89701 | *ycf1* | *IGS (rps7, trnL-CAA)* | 113733 | IRb, IRa | |  |
| 44 | 57 | P | 89719 | *ycf1* | *IGS (rps7, trnL-CAA)* | 113751 | IRb, IRa | |  |
| 45 | 57 | F | 113733 | *IGS (rps7, trnL-CAA)* | *IGS (rps7, trnL-CAA)* | 5113751 | IRa | |  |
| 46 | 53 | P | 7741 | *clpP* | *IGS (rps12, psbB)* | 67597 | LSC | |  |
| 47 | 53 | F | 65441 | *rps18* | *rps18* | 65576 | LSC | |  |
| 48 | 46 | F | 88490 | *IGS (trnL-CAA, ycf1)* | *IGS (trnL-CAA, ycf1)* | 88533 | IRb | |  |
| 49 | 46 | P | 88490 | *IGS (trnL-CAA, ycf1)* | *IGS (rps7, trnL-CAA)* | 114930 | IRb, IRa | |  |
