# Supplementary material for: Complete Chloroplast Genome of Rhipsalis baccifera, the only Cactus with Natural Distribution in the Old World: Genome Rearrangement, Intron Gain and Loss, and Implications for Phylogenetic Studies
Source: Plants (Basel). 2020 Jul 31;9(8):979. doi: 10.3390/plants9080979 (PMC7464518; doi:10.3390/plants9080979)
Supplement: Supplementary file 1 [file plants-09-00979-s001.zip › Table S4.docx]

**Table S4: Table showing the types of SSR repeats found in suborder Portulacineae**

|  | *T. paniculatum* | *M. zephyranthoides* | *M. albiflora* | *L. schottii* | *C. gigantea* | *M. solicioides* | *R. baccifera* |
| --- | --- | --- | --- | --- | --- | --- | --- |
| A/T | 66 | 31 | 56 | 41 | 29 | 65 | 35 |
| C/G | 2 | 0 | 1 | 0 | 0 | 2 | 3 |
| AG/CT | 1 | 0 | 0 | 1 | 1 | 0 | 0 |
| AT/AT | 11 | 7 | 10 | 9 | 9 | 14 | 9 |
| AC/GT | 0 | 1 | 0 | 0 | 0 | 0 | 0 |
| AAG/CTT | 0 | 2 | 3 | 0 | 0 | 3 | 2 |
| ACG/CGT | 0 | 1 | 0 | 0 | 0 | 0 | 0 |
| ACT/AGT | 0 | 0 | 0 | 0 | 0 | 0 | 1 |
| ATC/ATG | 0 | 2 | 0 | 0 | 0 | 0 | 0 |
| AGG/CCT | 0 | 0 | 1 | 0 | 0 | 2 | 0 |
| CCG/CGG | 0 | 0 | 0 | 1 | 0 | 0 | 2 |
| AAT/ATT | 5 | 0 | 1 | 1 | 1 | 2 | 0 |
| ACCT/AGGT | 2 | 0 | 0 | 1 | 1 | 0 | 1 |
| ACAG/CTGT | 0 | 0 | 0 | 0 | 0 | 0 | 1 |
| ACAT/ATGT | 0 | 0 | 0 | 0 | 0 | 0 | 1 |
| ATCC/ATGG | 1 | 0 | 0 | 0 | 0 | 0 | 1 |
| AATT/AATT | 2 | 0 | 2 | 0 | 0 | 1 | 2 |
| AAAG/CTTT | 1 | 3 | 3 | 1 | 1 | 1 | 1 |
| AAAC/GTTT | 0 | 0 | 0 | 1 | 1 | 0 | 0 |
| AAAT/ATTT | 0 | 1 | 2 | 3 | 0 | 3 | 0 |
| AAGG/CCTT | 1 | 1 | 1 | 0 | 0 | 0 | 1 |
| AATC/ATTG | 0 | 1 | 0 | 0 | 0 | 1 | 0 |
| AAATT/AATTT | 0 | 0 | 0 | 1 | 0 | 0 | 0 |
| AAAAC/GTTTT | 0 | 0 | 0 | 1 | 0 | 0 | 0 |
| AACAC/GTGTT | 0 | 2 | 0 | 0 | 0 | 0 | 0 |
| AAAGT/ACTTT | 0 | 0 | 0 | 1 | 0 | 1 | 0 |
| AATAT/ATATT | 0 | 0 | 0 | 0 | 0 | 2 | 0 |
| AAAAAT/ATTTTT | 0 | 1 | 0 | 0 | 0 | 0 | 0 |
| AAAAAG/CTTTTT | 0 | 0 | 0 | 1 | 0 | 1 | 0 |

| Table S4 continued | | | | | | | | |
| --- | --- | --- | --- | --- | --- | --- | --- | --- |
| ACGAGG/CCTCGT | | 0 | 1 | 1 | 0 | 0 | 0 | 0 |
| ACATAT/ATATGT | 0 | | 0 | 1 | 0 | 0 | 0 | 0 |
| AAGATC/ATCTTG | 0 | | 0 | 0 | 0 | 0 | 1 | 0 |
| AAATTG/AATTTC | 0 | | 0 | 0 | 0 | 0 | 0 | 2 |
| Total | 92 | | 54 | 82 | 63 | 43 | 99 | 62 |
